# Development of Bifunctional Three-Dimensional Cysts from Chemically Induced Liver Progenitors

**DOI:** 10.1155/2019/3975689

**Published:** 2019-09-03

**Authors:** Yu Huang, Yusuke Sakai, Takanobu Hara, Takeshi Katsuda, Takahiro Ochiya, Tomohiko Adachi, Masaaki Hidaka, Wei-Li Gu, Susumu Eguchi

**Affiliations:** ^1^Department of Surgery, Nagasaki University Graduate School of Biomedical Sciences, 1-7-1 Sakamoto, Nagasaki 852-8501, Japan; ^2^Department of Hepato-Pancreato-Biliary Surgery, Guangzhou First People's Hospital, School of Medicine, South China University of Technology, Guangzhou 510180, China; ^3^Department of Chemical Engineering, Faculty of Engineering, Graduate School, Kyushu University, 744 Motooka, Nishi-ku, Fukuoka 819-0395, Japan; ^4^Division of Molecular and Cellular Medicine, National Cancer Center Research Institute, 5-1-1 Tsukiji, Chuo-ku, Tokyo 104-0045, Japan

## Abstract

Chemically induced liver progenitors (CLiPs) have promising applications in liver regenerative medicine. Three-dimensional (3D) structures generated from liver progenitor cells possess wide applications in cell transplantation, disease model, and drug testing. Here, we report on the spontaneous formation of 3D cystic structures comprising maturing rat CLiPs on gelatin-coated dishes. Our 3D cysts contained Alb^+/+^CK19^+/−^ and Ck19^+/+^Alb^+/−^ cells. These cell types gradually diverged into specialized mature cells, as demonstrated by the expression of mature biliary markers (Cftr, Ae2, and Aqp1) and hepatic markers (Alb and Mrp2). The 3D cysts also expressed functional multidrug resistance protein 1 (Mdr1), as indicated by epithelial efflux of rhodamine. Furthermore, we observed bile canaliculi functions between hepatocytes and cholyl-lysyl-fluorescein extrusions, indicating that the functional characteristics of 3D cysts and active bile salt export pump (Bsep) transporters were intact. Thus, our study revealed a natural characteristic of rat CLiPs to spontaneously form 3D cystic structures accompanied with cell maturation *in vitro*, offering a platform for studies of liver development and drug screening.

## 1. Introduction

Several studies have shown that *in vivo* hepatocytes can transform into proliferative bipotent liver progenitor cells (LPCs) following chronic liver injury [1–4]. The *in vitro*-induced LPCs underwent transgene reprogramming era and now are stepping into the era of chemical reprogramming, which is a transgene-free approach that broke the limitation for clinical applications [5]. A recent study in Japan shows that the YAC cocktail of the three small molecules Y-27632, A-83-01, and CHIR99021 can convert mature rodent hepatocytes into proliferative bipotent cells *in vitro* which were previously identified as chemically induced liver progenitors (CLiPs) [6]. In Korea, researchers reprogrammed the human primary hepatocytes into hepatic progenitor cells by a combined treatment with two small molecules, A83-01 and CHIR99021, and hepatic growth factor (HGF) [7]. In China too, researchers have successfully converted the primary human hepatocytes into bipotent LPCs by using the three small molecules, Y-27632, A-83-01, and CHIR99021, and two growth factors, HGF and epidermal growth factor [8]. Those chemically induced LPCs have the potential to differentiate into hepatic or cholangiocytic cells *in vitro*. Furthermore, these cells were shown to extensively repopulate chronically injured liver tissues *in vivo* [6, 7] and can serve as a suitable model to study the host interactions with HBV and antiviral therapies [8].

With the development of stem cell techniques, several studies have established 3D structures, such as cysts, spheroids, and organoids, from LPCs or induced pluripotent stem cells (iPSCs) or embryonic stem cells (ESCs). In a study by Anzai et al. [9], they formed cystic structures with cholangiocytic characteristics from LPCs in an adult liver in a 3D culture system. Dianat et al. [10] developed cystic cholangiocytic structures from ESC/iPSC-derived hepatoblasts in a 3D matrix culture system. Similarly, Ogawa et al. [11] developed cystic and spheroid structures from iPSC-derived hepatoblasts cultured on collagen/Matrigel with OP9 stromal cells. Sampaziotis et al. [12, 13] also generated cholangiocyte-like cell organoids from iPSC-derived hepatoblasts and cholangiocytic progenitor cells. Wu et al. reported that they developed functional 3D hepatobiliary organoids generated form iPSCs in 45 days in a culture system [14]. Those above culture systems, however, have limitations such as either culturing in a 3D gel system, long culture days, or requirement for coculturing with other cells. No CLiPs were included in these reported researches either.

Here, we report about the spontaneous formation of CLiP-derived 3D cysts. Under the present culture conditions, rat CLiPs spontaneously generated 3D cysts with hepatic and cholangiocytic characteristics in gelatin-coated dishes. These 3D cysts comprised Alb^+/+^CK19^+/−^ and Ck19^+/+^Alb^+/−^ cells that functioned as mature hepatocytes and cholangiocytes, respectively. The formation of 3D cystic structures may be one of the native characteristics of CLiPs that allows maturation *in vitro.* Our study may facilitate the modeling of functional liver tissues in the era of chemical reprogramming.

## 2. Materials and Methods

### 2.1. Rats and Derivation of Hepatocytes

Male Wistar rats (age, 7–8 weeks; weight, 160–200 g; CLEA Japan Inc., Tokyo, Japan) were used. They were bred and housed at the rat facility. Animal handling was in accordance with the Guidelines for Animal Experimentation in Nagasaki University. Primary mature hepatocytes (MHs) were isolated by a modified two-step collagenase perfusion method in accordance with previous reports [15, 16]. Isolated cells were filtered through a cotton mesh membrane and a 45 *μ*m stainless mesh. They were then purified thrice by centrifugation at 50×g for 2 min each at 4°C. Cell suspensions were subsequently centrifuged with 40% Percoll PLUS solution (GE Healthcare, Tokyo, Japan) at 50×g for 20 min at 4°C. All experiments were performed using MHs with at least 90% viability, as determined using trypan blue exclusion tests. Isolation procedures were performed in Dulbecco's modified Eagle's medium (Wako Pure Chemical, Osaka, Japan) supplemented with 10% fetal bovine serum (FBS) (Life Technologies, Carlsbad, CA, USA), 10 mM 4-(2-hydroxyethyl)-1-piperazine ethanesulfonic acid (HEPES) (Dojindo, Kumamoto, Japan), 2 mM L-glutamine, 100 U/mL penicillin, and 100 mg/mL streptomycin (Life Technologies).

### 2.2. Reprogramming of MHs to CLiPs

Primary MHs were seeded onto collagen-coated dishes (Asahi Techno Glass, Tokyo, Japan) at a density of 1 × 10^4^ cells/cm^2^ in small chemical reprogramming culture medium as previously reported [6, 17]. The small chemical reprogramming culture medium was DMEM/F12 containing 2.4 g/L NaHCO_3_ and L-glutamine (Life Technologies) and supplemented with 5 mM HEPES, 30 mg/L L-proline, 0.05% BSA, 10 ng/mL epidermal growth factor (all from Sigma-Aldrich Japan, Tokyo, Japan), insulin-transferrin-serine (ITS)-X (Life Technologies), 10^−7^ M dexamethasone (Dex) (Fuji Pharma Co. Ltd., Tokyo, Japan), 10 mM nicotinamide (Sigma-Aldrich Japan), 1 mM ascorbic acid-2 phosphate (Wako Pure Chemical), 100 U/mL penicillin, and 100 mg/mL streptomycin (Life Technologies) in addition to 3 small chemical molecules of 10 *μ*M Y-27632 (AdooQ BioScience, Irvine, CA), 0.5 *μ*M A-83-01 (Wako Pure Chemical), and 3 *μ*M CHIR99021 (AdooQ BioScience) (YAC inhibitor cocktail). Culture media were changed 1 day after seeding and every two days thereafter. It takes 10–14 days to generate CLiPs from primary rat MHs. As an induction control, primary MHs were incubated in the above basal medium without YAC supplementation.

### 2.3. Generation of 3D Cysts

Following 10 min treatments with TrypLE Express (Life Technologies), CLiPs were harvested at 90%–100% confluence. CLiPs from different passages with or without cryopreservation were then seeded into gelatin-coated dishes (Asahi Techno Glass) at 3 × 10^4^ cells/cm^2^ and were incubated in the abovementioned small chemical reprogramming culture medium. On days 1–3 after seeding, cells were gently mixed with 5 mL pipettes without changing the culture medium. From day 4, cysts were collected into 15 mL tubes and were centrifuged at 7×g (200 rpm) for 2 min. Cyst pellets were then resuspended in fresh culture medium and were gently transferred into the original culture dishes. Culture medium was changed every 3–5 days thereafter. For comparison, CLiPs were seeded onto collagen-coated dishes under the conditions described above and media were replaced from the third day after seeding and every 3–5 days thereafter. Maximal numbers of cysts in one dish were achieved after seeding CLiPs at 1 × 10^5^ cells/cm^2^.

### 2.4. Collection of 3D Cysts

Three different methods were used to collect intact cysts for differing applications as follows: (i) All culture media containing cysts were gently collected into 15 mL tubes using a wide-bore pipette and were then centrifuged at 7×g (200 rpm) for 2 min to collect cyst pellets. (ii) All culture media containing the cysts were gently collected into 15 mL tubes using a wide-bore pipette. Tubes were then allowed to sit still for 10–15 min, and the precipitated cysts were collected from the bottom of the tube. (iii) Culture dishes with cysts were placed on the platform of the phase contrast microscope and were gently shaken to the left and right and then up and down. Cysts congregated in the middle of the dish and were aspirated in small medium volumes under the microscope using a wide-bore pipette. Methods (i) and (ii) were used to change media under clean bench conditions. Method (iii) was used to collect cysts after immunostaining washes.

### 2.5. Measurements of 3D Cyst Diameters

The longest diameters of cell cysts were automatically measured using phase contrast micrographs and NIS-Elements software (version 4.0, Nikon, Tokyo, Japan). Under live conditions, phase contrast images were taken with the same objective lens and diameters were measured using the length measurement mode of the microscope. Mean diameters were calculated in the same fields as average diameters. At least 30 cell cysts were measured in each field.

### 2.6. Gene Expression Analysis (Real-Time PCR)

Gene expression analyses were performed as previously described [16]. Samples were cultured in dishes under various conditions, and mRNA was extracted using a spin column (NucleoSpin RNA II; Macherey-Nagel, Düren, Germany). Synthesis of cDNA was performed using a high-capacity cDNA reverse transcription kit (Applied Biosystems, Tokyo, Japan). Samples were then stored at −20°C until polymerase chain reaction (PCR) analyses, which were performed using an Applied Biosystems StepOnePlus Real-time PCR System with TaqMan Gene Expression Assay Kits (Applied Biosystems; Supplementary [Supplementary-material supplementary-material-1]) according to the manufacturer's instructions. Briefly, PCR mixtures contained 1 *μ*L of cDNA, 0.5 *μ*L of TaqMan Gene Expression Assay probe, 5 *μ*L of TaqMan Fast Advanced Master Mix (both from Applied Biosystems), and 13.5 *μ*L of nuclease-free water. Thermocycling conditions were 95°C for 20 s followed by 40 cycles of 95°C for 1 s and 60°C for 20 s. Expression levels were quantified using the comparative cycle time method. Cycle threshold (Ct) values were automatically determined by the Applied Biosystems StepOnePlus Real-Time PCR System, and fold changes in gene expression were calculated by the 2^−ΔΔCT^ method. Expression levels were normalized to those of the housekeeping gene and internal control glyceraldehyde 3-phosphate dehydrogenase (*Gapdh*).

### 2.7. Immunostaining

Cultured cysts were fixed with 4% paraformaldehyde (PFA) in phosphate-buffered saline (PBS) (Wako Pure Chemical) for 10 min. Fixed samples were then incubated in 0.1% Triton X-100 (Sigma-Aldrich Japan) in PBS for 10 min and were blocked in PBS containing 1% BSA for 1 h at room temperature. The cysts were then incubated with primary antibodies diluted in PBS+1% BSA at 4°C overnight. After washing with PBS twice, they were incubated with appropriate secondary antibodies diluted in PBS+1% BSA for 2 h. All antibodies are listed in supplementary [Supplementary-material supplementary-material-1]. Nuclei were stained with 4′,6-diamidiono-2-phenylindole (DAPI) (DOJINDO) for 30 min. To avoid the loss of cysts during the washing steps, cysts were made to completely settle or were processed under the microscope. Washing times in PBS were up to 30–60 mins. Fluorescence images were captured using a confocal laser scanning microscope (TCS SP8; Leica Microsystems, Wetzlar, Germany).

### 2.8. Rhodamine 123 Staining

On day 14, 3D cysts were incubated with HBSS containing 100 *μ*M of rhodamine 123 (both from Sigma-Aldrich Japan) for 30 min at 37°C and were then washed with HBSS once. To inhibit multidrug resistance protein 1 (Mdr1) transporter activity, cysts were incubated with 20 *μ*M verapamil (Tokyo Chemical Industry Co. Ltd., Tokyo, Japan) at 37°C for 2 h before adding rhodamine 123. Stained cysts were visualized using a confocal microscope. Measurements of fluorescent intensity (FI) were made between the cyst interior and exterior using ImageJ software (https://imagej.nih.gov/ij/index.html). Rhodamine 123 fluorescence levels in 3D cyst lumens were normalized to background measurements in surrounding external areas. Numbers of cysts are reported in the figure legends. To make semiquantitative comparisons of inhibitor-induced changes, cysts were artificially sorted into four groups according to FI as follows: (a) strongly stained cysts (FI > 60), (b) moderately stained cysts (30 < FI ≤ 60), (c) weakly stained cysts (0 < FI ≤ 30), and (d) nonstained cysts (FI ≤ 0). Percentages of each cyst types are shown in a bar graph.

### 2.9. Cholyl-Lysyl-Fluorescein (CLF) Assays

Cysts were loaded with 1 *μ*M CLF (Corning Glass Works, Corning, NY, USA) for 30 min at 37°C and were then washed once with HBSS. Cysts were then observed and images were generated and captured using a confocal microscope. FI in measurements were determined as described above for rhodamine 123 staining assays.

### 2.10. Statistical Analysis

Statistical analyses were performed and graphs were made using GraphPad Prism 7.0 (GraphPad Software Inc., San Diego, CA, USA). Data are present as means ± standard deviations (s.d.). Details of statistical tests and the associated *p* values are described in the respective figure legends.

For extra details, see Supplemental Experimental Procedures in the Supplemental Information section.

## 3. Results

### 3.1. Reprogramed CLiPs Form 3D Cysts on Gelatin-Coated Dishes

As previously reported by Katsuda et al., passaged CLiPs are generally cultured on collagen-coated dishes in reprogramming medium containing 5% FBS for the first day after seeding, and without FBS thereafter [6]. FBS supplementation reportedly improves attachment of CLiPs to dishes. In these experiments, however, CLiPs cultured in gelatin-coated dishes without FBS congregated and floated in the medium ([Supplementary-material supplementary-material-1]). High magnification images showed the presence of cystic or spheroid structures after mechanical separation of the congregated cells using a pipette (Figures [Supplementary-material supplementary-material-1] and [Supplementary-material supplementary-material-1]). After several days without changing the culture medium, three-dimensional structures were formed spontaneously and were found floating in the culture medium.

**Figure 1:**
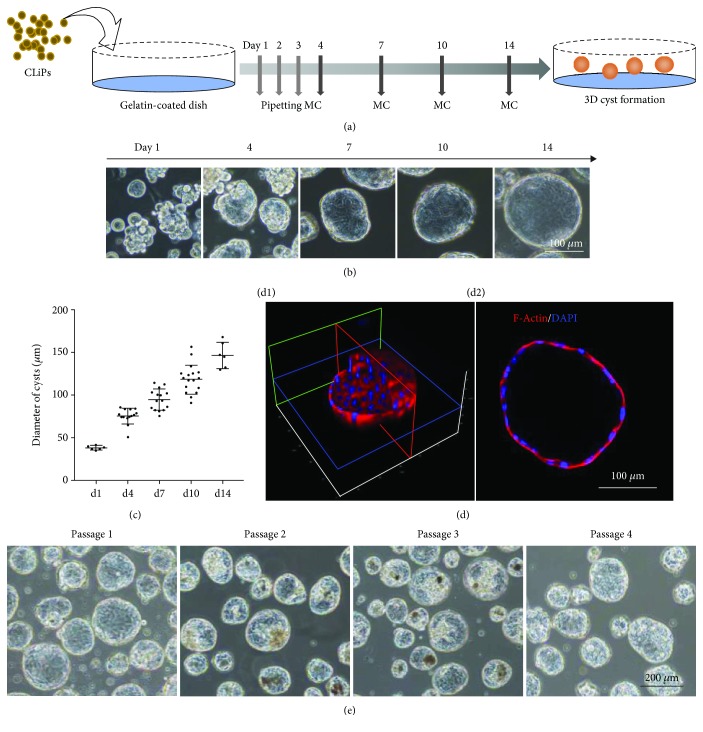
Reprogramed CLiPs form 3D cysts on gelatin-coated dishes (see also [Supplementary-material supplementary-material-1]). (a) Standardized protocol for inducing the formation of three-dimensional (3D) cysts from cultured CLiPs on gelatin-coated dishes; CLiPs were pipetted three times on each of the first three days, and culture medium change were replaced on days 4, 7, and 10. (b) Phase contrast images at indicated time points showing enlargement of 3D cysts; scale bar = 100 *μ*m. (c) Changes in cyst diameters at indicted time points; data are shown as the means ± standard deviations (s.d.; *n* ≥ 6), and more than 30 cysts were chosen for each experiment at all time points. (d) Immunostaining images of F-actin microfilaments in 3D cyst structures (d1) with hollow lumens (d2); scale bar = 100 *μ*m. (e) Phase contrast images of 3D cysts derived from CLiPs at various passages; scale bar = 200 *μ*m.

To standardize the protocol for spontaneous cyst formation, reprogramed CLiPs were incubated on gelatin-coated dishes in reprogramming medium without FBS for 2 weeks (Figure 1(a)). Because the CLiPs aggregated in the first few days, we pipetted them three times on each of these days and then changed the medium three times on days 4, 7, and 10 (Figure 1(a)). On day 14, 3D structures were formed and used in functional analyses. During two weeks of culture on gelatin-coated dishes, CLiPs formed floating 3D structures in the medium (Figure 1(b)). These 3D structures gradually grew and had average diameters of about 146.7 ± 28.7 *μ*m on day 14 (Figure 1(c)). Control CLiPs were also cultured on collagen-coated dishes and only formed monolayers ([Supplementary-material supplementary-material-1]). To investigate the morphology of structures with and without internal lumen, we performed immunohistochemical staining of F-actin microfilaments. The resulting data showed that our 3D structures have hollow lumens (Figure 1(d)) that differed from spheroid structures without hollow lumens, indicating the formation of cystic structures. CLiPs from passages P1–P4 could form 3D cysts (Figure 1(e)) and be cultured for longer than 1 month ([Supplementary-material supplementary-material-1]). Cryopreserved CLiPs could also form 3D cysts under the same conditions ([Supplementary-material supplementary-material-1]).

### 3.2. Spontaneously Formed 3D Cysts Are Biphenotypic

**Figure 2:**
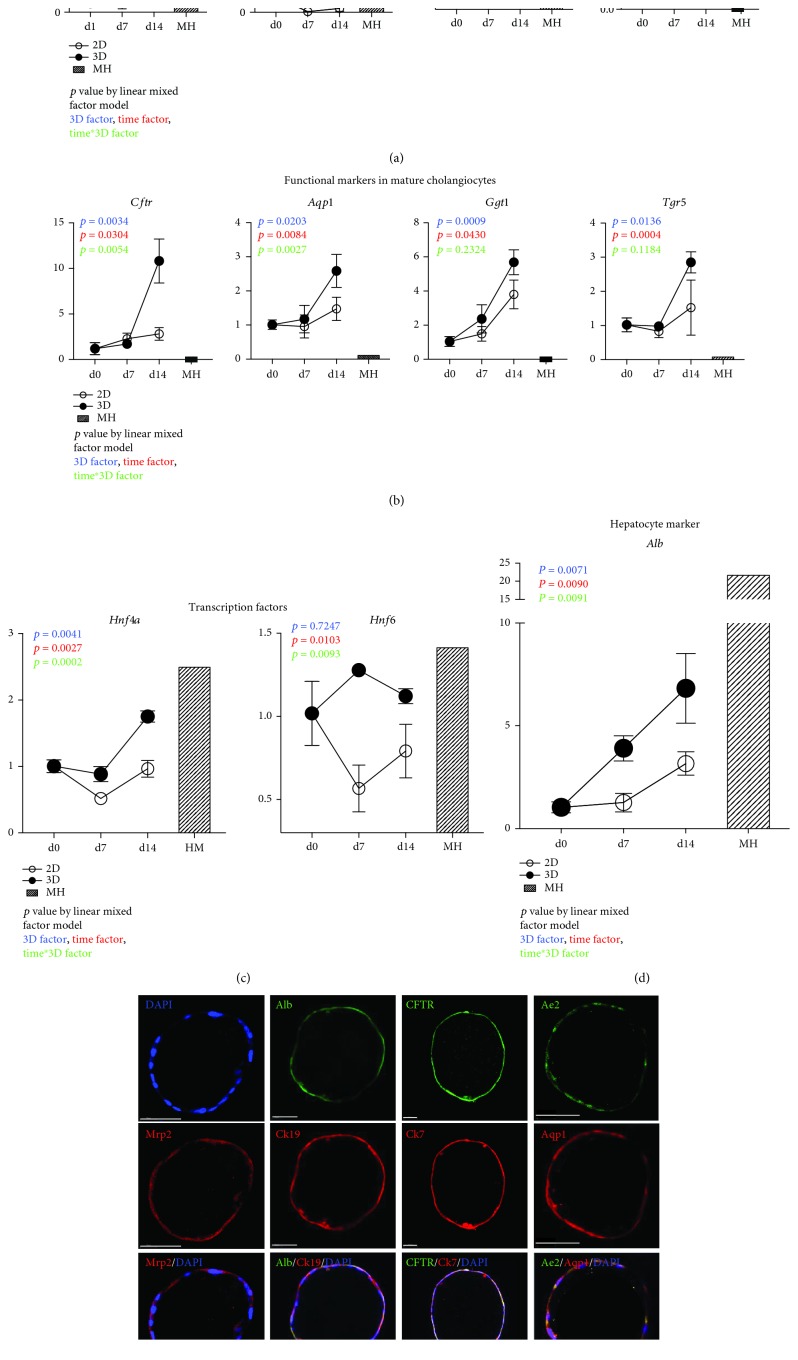
Spontaneously formed 3D cysts are biphenotypic (see also [Supplementary-material supplementary-material-1]). (a) Time course expression profiles of hepatic uptake transporter (*Oatp2* and *Pept1*) and efflux transporter (*Mrp2* and *Bsep*) genes in samples from 3D cysts and 2D monolayer cultures were assessed using quantitative reverse transcriptase-polymerase chain reaction (qRT-PCR) analyses. (b) Time course expression profiles of functional marker expression levels in mature cholangiocytes (*Cftr*, *Aqp1*, *Tgr5*, and *Ggt1*) from samples of 3D cysts and 2D cell monolayers were assessed using qRT-PCR analysis. (c) Time course of expression levels of the hepatic transcription factors *Hnf4a* and *Hnf6* in samples from 3D cyst and 2D monolayer cells were assessed using qRT-PCR analyses. (d) Time course expression profiles of hepatic marker *Albumin* (*Alb*). Data are shown as means ± s.d. of three experiments. Values were normalized to those of *Gapdh* and are presented as fold changes relative to an expression level of 1 at day 0; the primary mature hepatocytes (MH) were also labeled. *p* values were calculated using a linear mixed model to account for the covariant structures that follow repeated measures at different time points. The color descriptors are described in the figure. Differences were identified using two-way ANOVA. (e) Immunocytochemical analyses of 3D cysts at day 14 showing expression levels of the epithelial cell markers Ck19 and Ck7, the hepatic marker albumin (Alb), the hepatocyte transporter Mrp2, and the mature cholangiocytic markers CFTR, Aqp1, and Ae2 (see also the supplemental videos for details).

To analyze the characteristics of 3D cysts, we compared expression levels of hepatobiliary marker genes at indicated times in samples from two-dimensional (2D) monolayer cells on collagen-coated dishes. The quantitative result that showed the upregulation of hepatocyte membrane transporters (Figure 2(a)) in 3D cysts included organic anion transmembrane transport 2 (*Oatp2*) (also known as *Slco1b1*), peptide transporter 1 (*Pept1*) (also known as *Slc15a1*), multidrug resistance-associated protein 2 (*Mrp2*) also known as *Abcc2*), the bile salt export pump (*Bsep*) (also known as *Abcb11*), and *Albumin* (*Alb*) (Figure 2(d)), indicating hepatic characteristics of 3D cyst. Simultaneously, the upregulation of mature biliary markers occurred in 3D cysts on day 14 as well, including the cystic fibrosis transmembrane conductance regulator (*Cftr*), G protein-coupled bile acid receptor 1 (*Gpbar*) (also known as *Tgr5*), gamma-glutamyl-transferase 1 (*Ggt1*) (also known as *CD224*), and aquaporin 1 (*Aqp1*) (Figure 2(b)), demonstrating the biliary characteristics of 3D cysts. Furthermore, the transcript factors Hnf4a, which controls the onset of hepatocyte cell fates [18], and Hnf6, which is a key factor of normal bile duct development [19–21], were also upregulated in 3D cysts at comparable levels in primary mature hepatocyte (Figure 2(c)).

Considering the expression of hepatic and biliary gene markers in 3D cysts, we performed immunocytochemical analyzes of protein markers of hepatic and biliary functions in 3D cysts. These analyses revealed the presence of epithelial-like cells that express cytokeratin-19 (Ck19) (also known as Krt19) and Ck7 (also known as Krt7) in 3D cysts after 14-days culture (Figures 2(e) and [Supplementary-material supplementary-material-1]). Concomitantly, these cysts expressed the mature hepatic markers albumin (Alb) and the hepatocyte transporter Mrp2 and the mature cholangiocytic markers CFTR, Aqp1, and anion exchange protein 2 (Ae2), indicating the bipotent functionality of 3D cysts following *in vitro* maturation (Figures 2(e) and [Supplementary-material supplementary-material-1]). See also the multilayers of stained cyst in supplementary videos [Supplementary-material supplementary-material-1]–[Supplementary-material supplementary-material-1]. Collectively, these data show that the spontaneously formed 3D cysts with hepatic and biliary cell gene expression profiles are biphenotypic in nature.

### 3.3. Hepatocytes and Cholangiocytes Are Randomly Distributed in 3D Cysts

**Figure 3:**
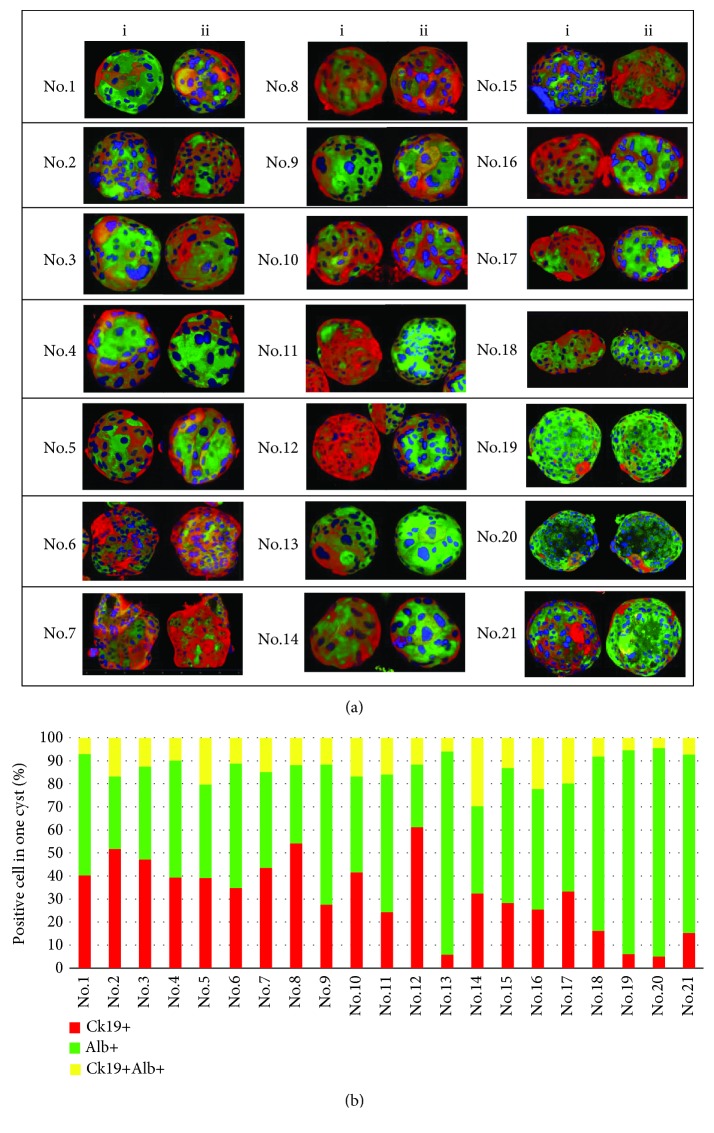
Distributions of hepatocytes and cholangiocytes in 3D cysts (see also [Supplementary-material supplementary-material-1]). (a) Confocal microscopy images of 21 cysts stained with antibodies against Ck19 and Alb; single cyst was split into two spheroids; (i) and (ii) represent superior and inferior spheroids, respectively. (b) Percentages of Ck19^+^ (red), Alb^+^ (green), and Ck19^+^Alb^+^ (yellow) cells in 21 cysts.

As shown above, 3D cysts have biphenotypic cell compositions of hepatocytes and cholangiocytes. Thus, we analyzed distributions of hepatocytes and cholangiocytes in single cysts using the hepatocyte and cholangiocyte markers Alb and Ck19, respectively, and acquired three-dimensional images from *x*-, *y*-, and *z*-stack observations ([Supplementary-material supplementary-material-1]). To visually describe cyst compositions, we split a single cyst into superior (a) and inferior (a') spheres (Figure 3(a)) or performed image analyzes of a single cyst as a cube with six aspects ([Supplementary-material supplementary-material-1]). Our data show that single cysts comprise cells with high levels of Alb and low levels of Ck19 and other cells expressing high levels of Ck19 and low levels of Alb, confirming the biphenotypic cell composition of 3D cysts (Figure 3(a)). In immunostaining analyses of twenty-one cysts (Figure 3(b)), hepatocytes and cholangiocytes were distributed randomly but some were hepatocyte based (nos. 13, 19, and 20) and others were cholangiocyte based (nos. 2, 8, and 12). These arrangements support our theory of biphenotypic potency of maturing CLiPs.

### 3.4. Spontaneously Formed 3D Cysts Are Functional

**Figure 4:**
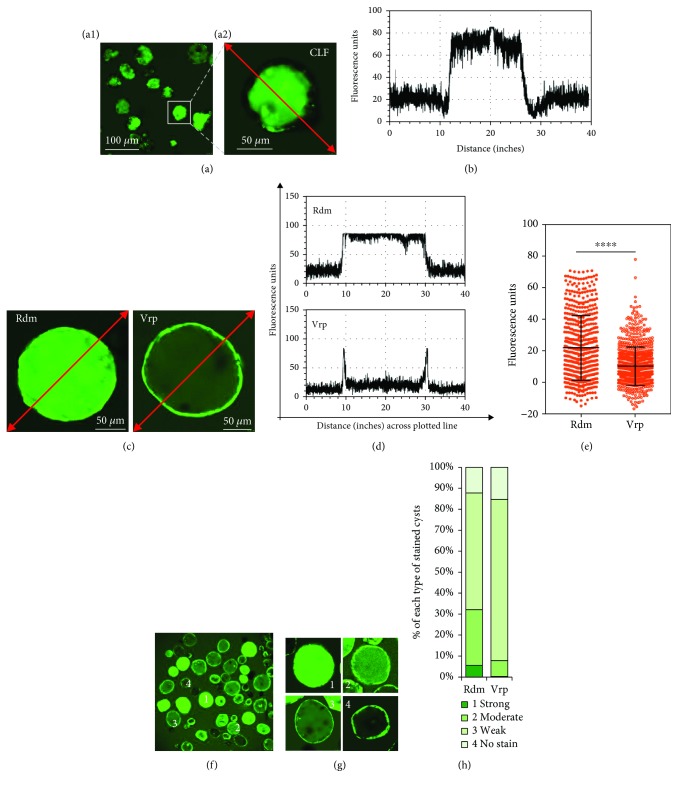
Spontaneously formed 3D cysts are functional (see also [Supplementary-material supplementary-material-1]). (a) Representative fluorescence images at low magnification (a1) and high magnification (a2) demonstrate active transport of the fluorescent bile acid cholyl-lysyl-fluorescein (CLF) into the lumens of cysts. (b) Fluorescence intensity (FI) measurements along the red line in images from (a). (c) Representative fluorescence images demonstrating secretion of fluorescent rhodamine 123 (Rdm) into cyst lumens; secretions were inhibited by the Mdr1 inhibitor verapamil (Vrp), thus confirming that Mdr1 is functional. (d) FI measurements along the red line in the images from (c). (e) Mean intraluminal FI was normalized to background fluorescence in the absence (Rdm) or presence (Vrp) of verapamil; *n*_(Rdm)_ = 573, *n*_(Vrp)_ = 765 measurements. Data are shown as means ± s.d.; ^∗∗∗∗^*P* < 0.0001 (two-tailed Mann-Whitney *U* test). (f) A representative low-magnification fluorescence image with varying FI in the lumens of cysts 1, 2, 3, and 4. (g) High-magnification image of that shown in (f); 1: strongly stained cyst (FI > 60); 2: moderately stained cyst (30 < FI ≤ 60); 3: weakly stained cyst (0 < FI ≤ 30); 4: nonstained cyst (FI ≤ 0). (h) The bar graph of percentages of the four types of stained cysts demonstrates that Mdr1 function in cysts is inhibited by verapamil.

**Figure 5:**
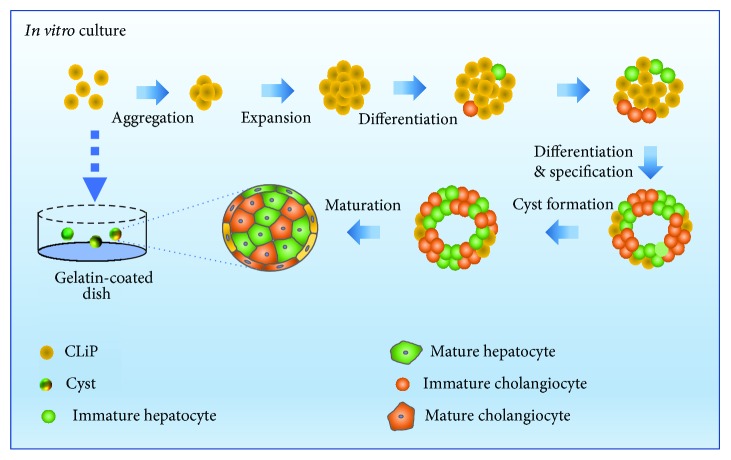
Overview of spontaneous formation of 3D cysts *in vitro* for rat CLiPs as a course for maturation.

Because the present 3D cysts expressed mature hepatic and biliary markers, we performed functional analyzes. Initially, the capacity of 3D cysts to transport bile acids through the Bsep was tested according to accumulations of the fluorescent bile acid CLF inside the central lumen (Figures 4(a) and 4(b)). CLF-stained cysts in culture were chronologically found as indication for cystic maturation with expression of transporter Bsep ([Supplementary-material supplementary-material-1]). Yet, some cysts were not stained nor had low CLF staining, indicating that compositions of 3D cysts vary. Subsequently, we evaluated rhodamine 123 efflux from 3D cysts. This dye has been used to measure Mdr1 transporter activity in normal bile duct cells and progenitor cells [22, 23]. 3D cysts transported rhodamine into the luminal space, suggesting the presence of active transporters (Figures 4(c) and 4(d) and [Supplementary-material supplementary-material-1]). Moreover, treatment with the Mdr1 inhibitor verapamil prevented accumulation of rhodamine in the lumen of most cysts, confirming that observed dye movements reflect active transport through Mdr1 (Figures 4(c) and 4(d) and [Supplementary-material supplementary-material-1]). In further experiments, we compared mean intraluminal FI normalized to the background in the absence (Rdm) and presence (Vrp) of verapamil. Verapamil strongly suppressed dye transport into the cystic lumen (*p* < 0.0001, two-tailed Mann-Whitney *U* test; Figure 4(e)). In addition to the biphenotypic cell properties of 3D cysts described above, the capacity to transport rhodamine varied between cysts (Figures 4(f) and 4(g)). Accordingly, we sorted 3D cysts into four categories according to relative mean intraluminal FI normalized to respective background measurements (Figures 4(f) and 4(g)) and showed that the Mdr1 inhibitor verapamil alters percentages and distributions of the four stained types (Figures [Supplementary-material supplementary-material-1] and [Supplementary-material supplementary-material-1]). The Mdr1 inhibitor, verapamil, can change the percentage and distributions of those four stained types (Figure 4(h)). Compared with the no inhibition group (Rdm), the Mdr1 inhibitor can reduce about 95.3% of the strongly stained cyst and 71.4% of the moderately stained cyst and can increase 38.1% of the weakly stained cyst and 25.2% of the unstained cyst in detail (Figure 5(h)). These observations confirm that spontaneously formed 3D cysts display functions of hepatic and biliary epithelial tissues.

## 4. Discussion

Chemical reprogramming technique offers us a new approach for generation of bipotent LPCs from mature hepatocytes. Compared with the transgene methods, chemical reprogramming approach has broadened the clinical application of the induced LPCs as they do not require an exogenous virus. As a new dawn of the chemical reprogramming technique, there are many aspects that can be illuminated, such as *in vitro* culture characteristics. In the present work, we generated functional 3D cystic structures from CLiPs under comparatively natural culture conditions in gelatin-coated dishes. We show that 3D cysts generate from CLiPs independently of 3D gel culture technologies, exogenous cells, and genetic manipulations, offering a new platform for studies on liver development and drug screening as well as offering the potential opportunity to rebuild heteroaggregate liver organs using chemical reprogramming technique. Our spontaneously formed cysts without fixation in the culture medium also have the advantage, that is, they can float in the medium. It will facilitate the testing of drugs in culture medium, with greater access of chemicals to cells than in structures generated using a gel embedding system.

Although the ESC/iPSC-derived 3D structures were reportedly established in several studies, these culture systems, however, were either cultured in a gel embedding system with long culture days or by coculturing with other cells [9–11, 14]. In the present work, we found that the chemically reprogramed bipotent LPCs spontaneously developed into a 3D cystic structure with a lumen inside of them. To the best of our knowledge, in the free (i.e., no embedding) culture condition, these spontaneous generation characteristics are not present in ESCs or iPSCs; thus, we speculate that it is a specialty of the CLiPs. We contend that rat CLiPs naturally form 3D cysts and that this process may be more easily achieved using other methods through which the attachment of CLiPs to culture dishes is reduced, such as the hanging drop and rotary cell culture systems.

Because LPCs have the potential to transdifferentiate into hepatocytes or cholangiocytes for maturation in special culture medium, we speculated if the spontaneous formation of the 3D cystic structure is a course of self-maturation *in vitro*. Followed by analyzing cell types in our 3D cystic structures, initially using genetic profiles, we found that both hepatic and biliary genes were upregulated in 3D cysts, suggesting the possibility that spontaneously formed 3D cysts could mature into both hepatic and biliary epithelial tissues. Correspondingly, histologic staining analyzes of the hepatic marker albumin and the biliary marker CK19 supported our observations of biphenotypic cell compositions of this model, with Alb^++^CK19^+/−^ and CK19^++^Alb^+/−^ cells being identified in single cysts. We also observed positive staining of other functional biliary markers such as CFTR, Ae2, and Aqp1 and the functional hepatic marker Mrp2, suggesting the hepatic and biliary maturation of cells in 3D cysts. We speculate that formation of 3D structures is indicative of maturation of the chemically reprogrammed LPCs.

Biphenotypic characteristic usually implies bifunctionality, which is based on the expression of mature hepatocyte and cholangiocyte marker proteins. Specifically, functional Mdr1 transporters were identified in 3D cysts, indicting the presence of physiologically relevant biliary cells. This protein is known to be expressed on the apical membranes of mature bile ducts and on luminal membranes of cholangiocytes [22–24]. The hepatocyte factor CLF was also present inside the central lumen of 3D cysts, further demonstrating function transport through Bsep, which is expressed as a part of the canalicular system in hepatocytes [25, 26]. These functional data were also consistent with qRT-PCR analyses. Although levels of some hepatic genes are lower to those in primary hepatocytes, the levels of biliary genes, in contrast, are highly upregulated even when compared with those in primary hepatocytes. Thus, our analyses of cell functions further indicated that CLiPs mature into hepatic and biliary cells in our culture conditions.

In contrast with the cells from 2D monolayer cultures, our spontaneously formed 3D cysts expressed transcripts for transporters and proteins with bold folds, including Mrp2, Otap2, CFTR, Gpbar, and Ggt1, which are known to be the transporters of bile salts [27] and organic anions [28]. These data suggest further application of 3D cysts. For example, CFTR, which detects no expression in primary hepatocytes in rats, was expressed at 5–10-fold higher levels in 3D structures, which resembled structures observed in patients with cystic fibrosis [29, 30]. With these morphological and genetic characteristics, 3D cysts have potential uses in the validation of novel therapeutic drugs [11, 13]. Studies have been planned to investigate this application of the present 3D cysts.

It is an important issue to apply CLiP to drug discovery and regenerative medicine in the future. Study of liver regenerative therapy using CLiP for liver cirrhosis and nonalcoholic steatohepatitis is permitted by the Japanese government lunched by our groups. In the case of iPSC cells, application researches for drug discovery and regenerative medicine are already in progress [31–33]. In a recently published work, Ouchi et al. [34] developed a reproducible method to generate multicellular human liver organoids from iPSCs and ESCs. Those iPSC-derived organoids recapitulate progressive features of steatohepatitis, including steatosis, inflammation, and fibrosis. Furthermore, the patient-derived organoid with lysosomal acid lipase deficiency exhibits the exaggerated steatohepatitis phenotype, as seen in vivo, and can be rescued by FGF19 [34]. This kind of research map of patient-derived iPSCs in combination with our 3D cystic culture system would further broaden our pathways to test the drugs for aforementioned cystic fibrosis disease.

Functional livers comprise multiple cell types, in which hepatocyte and biliary cells contribute the most [35, 36]. Liver organ or hepatobiliary organoid generation *in vitro* requires either a coculture of hepatocytes with other cells or the use of complex culture systems with hepatic and biliary differentiation medium [14]. We directly cultured CLiPs to form 3D cysts with hepatocytes and biliary cells. This fact makes it possible to rebuild heteroaggregate hepatobiliary organoids comprising two cell types following differentiation from a single LPC type. Besides this, future analyses to identify key factors that regulate the formation of homogeneous CLiP-derived 3D cysts with cholangiocytic functions will improve the establishment of *in vitro* bile ductal disease models. Simultaneously, homogeneous CLiP-derived 3D cysts with hepatic function comprising only hepatocytes may also provide therapeutic applications.

## 5. Conclusions

In summary, we show that 3D cysts readily and spontaneously form heterogeneous populations in gelatin-coated dishes (Figure 5). We contend that these morphological qualities are characteristic of rat CLiPs. Hence, our findings provide insights into the morphological characteristics of CLiPs and suggest strategies for the control of cell morphology in cultures on coated basements. Our results indicate new experimental avenues for modeling of liver development and morphogenesis during progenitor cell maturation. In particular, we show a potential method for assembling heteroaggregate hepatobiliary organoids comprising two cell types that differentiated from a single LPC type.

## Data Availability

The data used to support the findings of this study are included within the article.

## Supplementary Materials

Supplementary 1 Figure S1 (related to Figure 1): chemically induced liver progenitors (CLiPs) floated in culture medium on gelatin-coated dishes. (a) Cell morphology of CLiPs at day 1 after seeding onto gelatin-coated dishes before pipetting; images show huge cell aggregates floating on the medium. (b) The low magnification image of cell morphology after pipetting shows small cell aggregates as cell spheroids or cell cysts. (c) A high-magnification image of the aggregates after pipetting. (d) Cell morphology of control CLiPs at days 1 and 7 after seeding onto collagen-coated dishes. (e) Cell morphologies of CLiPs at days 20 and 30 after seeding onto collagen-coated dishes. (f) Cell morphologies of cryopreserved CLiPs at day 14. Figure S2 (related to Figure 2): high-magnification immunostaining of 3D cysts. Confocal microscopy images of immunostained histological sections of 3D cysts at day 14 show expression of the epithelial cell markers Ck19 and Ck7, the hepatic marker albumin (Alb), the hepatocyte transporter Mrp2, and the mature cholangiocytic markers CFTR, Aqp1, and Ae2. Figure S3 (related to Figure 3): six viewing angles of a single cyst. (a) A three-dimensional confocal image of cyst no. 21 from Figure 4 stained with albumin (green) and CK19 (red). (b) Six viewing angles of a cyst showing Alb^++^CK19^+/−^ and CK19^++^Alb^+/−^ cells: (1) superior aspect; (2) anterior aspect; (3) inferior aspect; (4) right lateral aspect; (5) posterior aspect; (6) right lateral aspect; (1') and (3') show the internal views of (1) and (3), respectively. Figure S4 (related to Figure 4): spontaneously formed 3D cysts are functional. (a) Confocal microscopy images of CLF-stained 3D cysts stained on days 1, 4, 7, 10, and 14; chronologically enhanced CLF-stained cysts during culture demonstrated the maturation of those cysts with the transporter Bsep. (b) Representative three-dimensional view of cysts exporting rhodamine 123 dye in the absence (left panel) and presence (right panel) of the Mdr1 inhibitor verapamil; the rhodamine 123 transporting function of Mdr1 in cysts was inhibited. (c) Representative fluorescence images with varying FI in the lumen of cysts in the absence of verapamil; 1: strongly stained cyst; 2: moderately stained cyst; 3: weakly stained cyst; 4: unstained cyst; relative mean intraluminal FI was normalized to respective background measurements. The right panel shows a histogram of cyst numbers at the different ranges of FI, indicating changed distributions of FI in cysts due to the presence of the Mdr1 inhibitor verapamil. The gram was set using Microsoft Excel according to the data series of FI. Red lines show curves of the distribution changes. (d) Representative fluorescence image showing varying FI in the lumens of cysts in the presence of verapamil; 1: strongly stained cyst; 2: moderately stained cyst; 3: weakly stained cyst; 4: unstained cyst; relative mean intraluminal FI was normalized to respective background measurements. The right panel shows a histogram of cyst numbers in the different ranges of FI, indicating changed distributions of FI in cysts due to the presence of the Mdr1 inhibitor, verapamil. The gram was set using Microsoft Excel according to the data series of FI. Red lines show curves of the distribution changes. Table S1: TaqMan gene expression assay numbers for real-time PCR analysis. Table S2: list of antibodies used for immunocytochemistry analyses.

Supplementary 2 Video 1: cyst stained with Ae2 (red)+Alb (yellow)+DAPI (blue).

Supplementary 3 Video 2: cyst stained with Aqp1 (red)+Alb (yellow)+DAPI (blue).

Supplementary 4 Video 3: cyst with CFTR (red)+Alb (yellow)+DAPI (blue).

Supplementary 5 Video 4: cyst with Krt19 (red)+Alb (green)+DAPI (blue).

Supplementary 6 Video 5: cyst with Krt19 (red)+Alb (yellow)+DAPI (blue).
