# Supplementary material for: Development of Bifunctional Three-Dimensional Cysts from Chemically Induced Liver Progenitors
Source: Stem Cells Int. 2019 Sep 3;2019:3975689. doi: 10.1155/2019/3975689 (PMC6745155; doi:10.1155/2019/3975689)
Supplement: Supplementary 1 — Figure S1 (related to Figure 1): chemically induced liver progenitors (CLiPs) floated in culture medium on gelatin-coated dishes. (a) Cell morphology of CLiPs at day 1 after seeding onto gelatin-coated dishes before pipetting; images show huge cell aggregates floating on the medium. (b) The low magnification image of cell morphology after pipetting shows small cell aggregates as cell spheroids or cell cysts. (c) A high-magnification image of the aggregates after pipetting. (d) Cell morphology of control CLiPs at days 1 and 7 after seeding onto collagen-coated dishes. (e) Cell morphologies of CLiPs at days 20 and 30 after seeding onto collagen-coated dishes. (f) Cell morphologies of cryopreserved CLiPs at day 14. Figure S2 (related to Figure 2): high-magnification immunostaining of 3D cysts. Confocal microscopy images of immunostained histological sections of 3D cysts at day 14 show expression of the epithelial cell markers Ck19 and Ck7, the hepatic marker albumin (Alb), the hepatocyte transporter Mrp2, and the mature cholangiocytic markers CFTR, Aqp1, and Ae2. Figure S3 (related to Figure 3): six viewing angles of a single cyst. (a) A three-dimensional confocal image of cyst no. 21 from Figure 4 stained with albumin (green) and CK19 (red). (b) Six viewing angles of a cyst showing Alb++CK19+/− and CK19++Alb+/− cells: (1) superior aspect; (2) anterior aspect; (3) inferior aspect; (4) right lateral aspect; (5) posterior aspect; (6) right lateral aspect; (1') and (3') show the internal views of (1) and (3), respectively. Figure S4 (related to Figure 4): spontaneously formed 3D cysts are functional. (a) Confocal microscopy images of CLF-stained 3D cysts stained on days 1, 4, 7, 10, and 14; chronologically enhanced CLF-stained cysts during culture demonstrated the maturation of those cysts with the transporter Bsep. (b) Representative three-dimensional view of cysts exporting rhodamine 123 dye in the absence (left panel) and presence (right panel) of the Mdr1 inhibitor ve [file 3975689.f1.docx]

**Supplementary Material**

**Supplemental Figures and Legends**

**
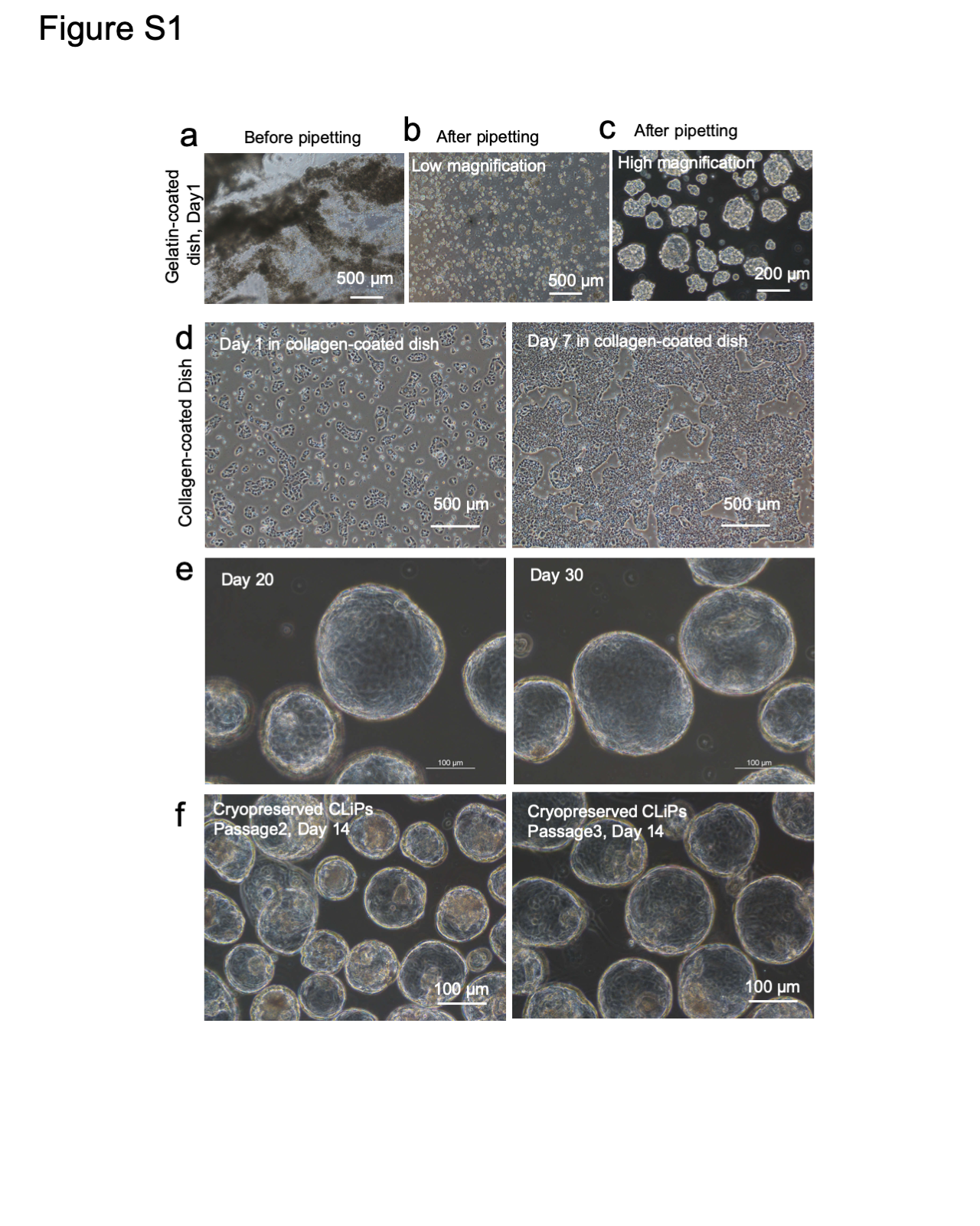
**

**Figure S1** (related to Figure 1). Chemically induced liver progenitors (CLiPs) floated in culture medium on gelatin-coated dishes.

(a). Cell morphology of CLiPs at day 1 after seeding onto gelatin-coated dishes before pipetting; images show huge cell aggregates floating on the medium.

(b). The low magnification image of cell morphology after pipetting shows small cell aggregates as cell spheroids or cell cysts.

(c). A high magnification image of the aggregates after pipetting.

(d). Cell morphology of control CLiPs at days 1 and 7 after seeding onto collagen-coated dishes

(e). Cell morphologies of CLiPs at days 20 and 30 after seeding onto collagen-coated dishes

(f). Cell morphologies of cryopreserved CLiPs at day 14

**
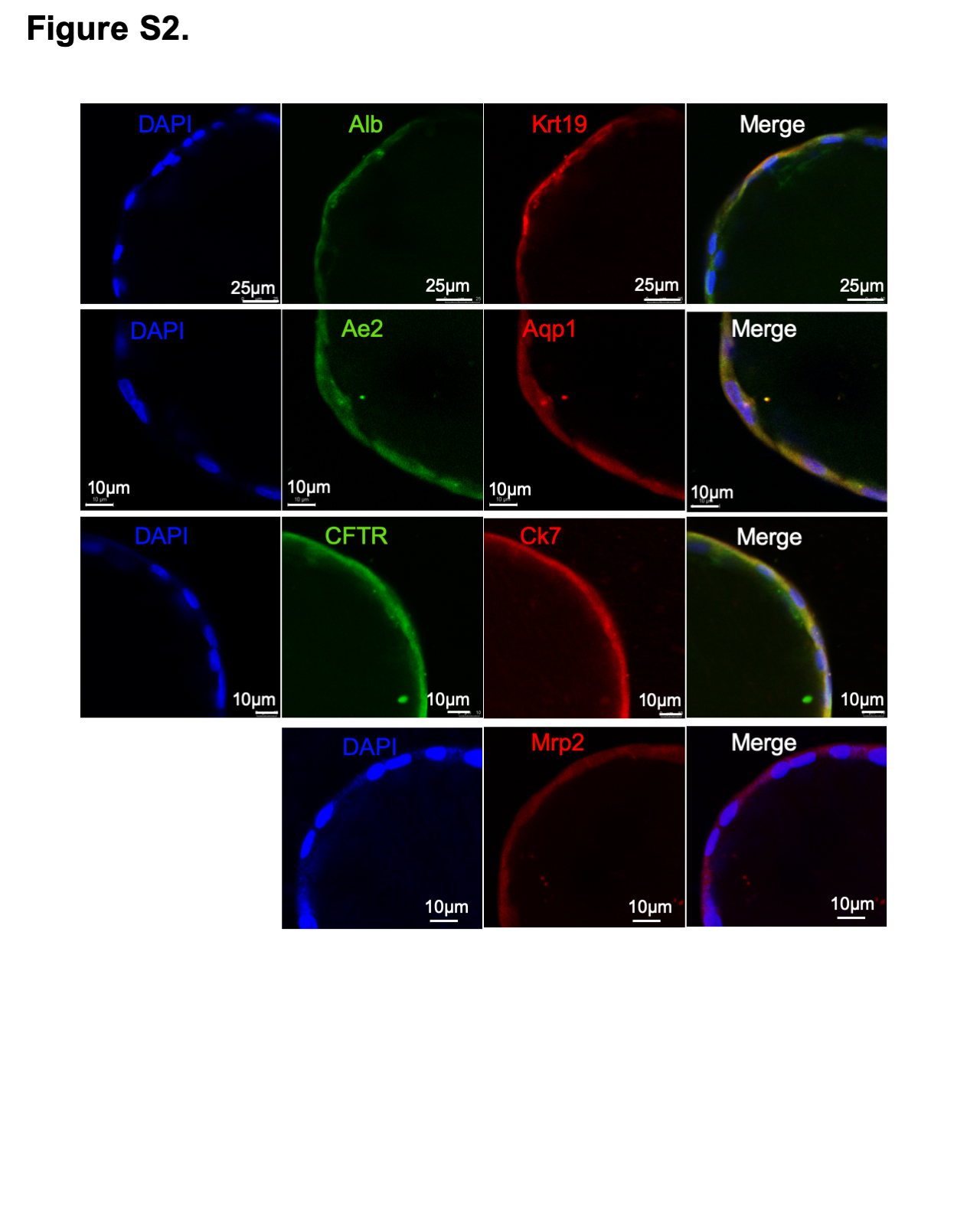
**

**Figure S2** (related to Figure 2). High magnification immunostaining of 3D cysts

Confocal microscopy images of immunostained histological sections of 3D cysts at day 14 show expression of the epithelial cell markers Ck19 and Ck7, the hepatic marker albumin (Alb), the hepatocyte transporter Mrp2, and the mature cholangiocytic markers CFTR, Aqp1, and Ae2.

**
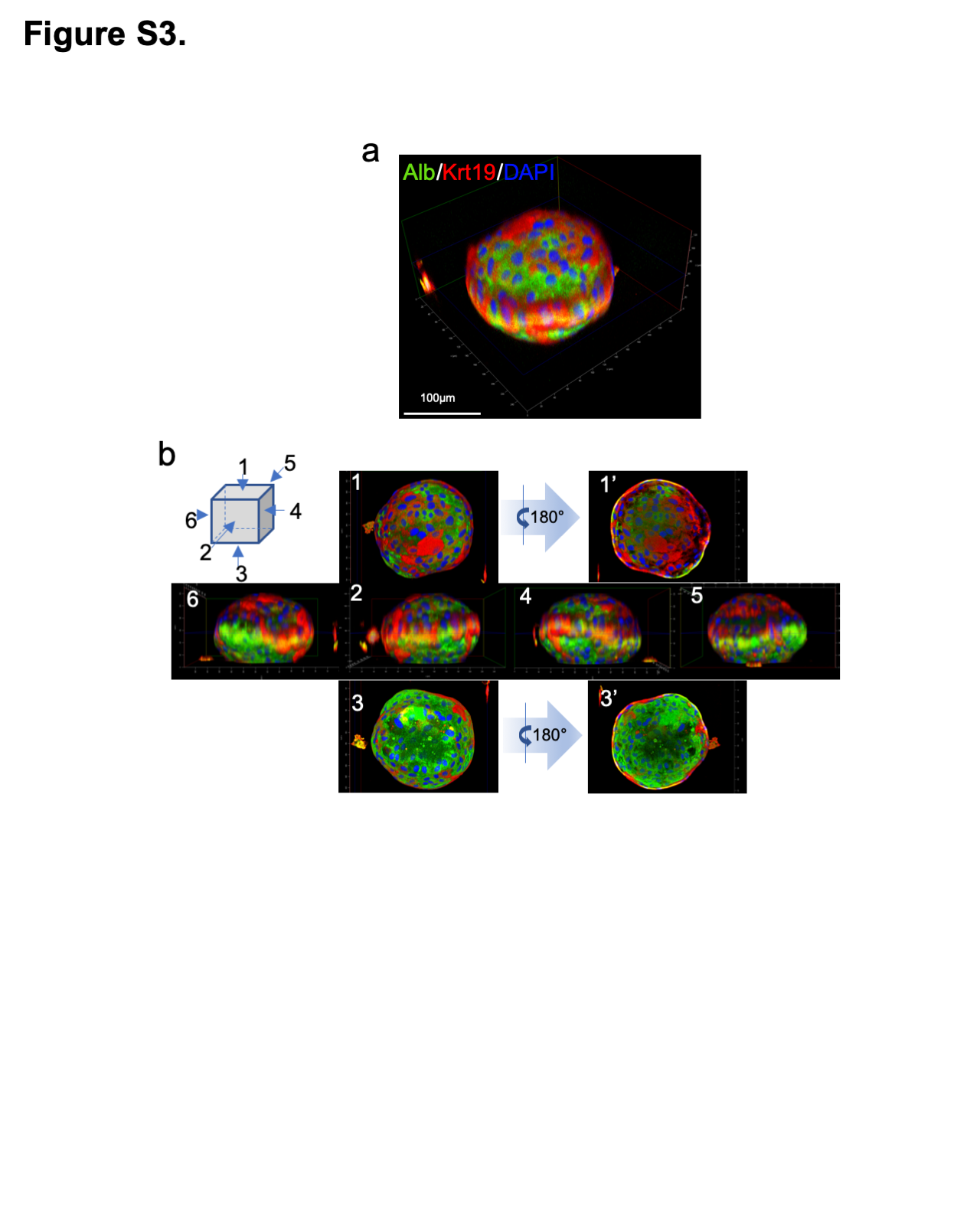
**

**Figure S3** (related to Figure 3). Six view angles of a single cyst.

(a) A three-dimensional confocal image of cyst No 21 cyst from Figure 4 stained with albumin (green) and CK19 (red)

(b) Six angles of view of a cyst showing Alb^++^CK19^+/-^ and CK19^++^Alb^+/-^ cells; (1), superior aspect; (2), anterior aspect; (3), inferior aspect; (4), right lateral aspect; (5), posterior aspect; (6), right lateral aspect; (1’) and (3’) show the internal views of (1) and (3), respectively.

**Figure S4** (related to Figure 4); Spontaneously formed 3D-cysts are functional.

(a) Confocal microscopy images of CLF stained 3D-Cysts stained on days 1, 4, 7, 10 and 14; chronologically enhanced CLF-stained cysts during culture demonstrated the maturation of those cysts with the transporter Bsep.

(b) Representative three-dimensional view of cysts exporting rhodamine 123 dye in the absence (left panel) and presence (right panel) of the Mdr1 inhibitor verapamil; the rhodamine 123 transporting function of Mdr1 in cysts was inhibited.

(c) Representative fluorescence images with varying FI in the lumen of cysts in absence of verapamil; 1, strongly stained cyst; 2, moderately stained cyst; 3, weakly stained cyst; 4, non stained cyst; relative mean intraluminal FI were normalized to respective background measurements. The right panel shows a histogram of cyst numbers at the different ranges of FI, indicating changed distributions of FI in cysts due to the presence of the Mdr1 inhibitor verapamil. The gram was set using Microsoft Excel according to the data series of FI. Red lines show curves of the distribution changes.

(d) Representative fluorescence image showing varying FI in the lumens of cysts in presence of verapamil; 1, strongly stained cyst; 2, moderately stained cyst; 3, weakly stained cyst; 4, non stained cyst; relative mean intraluminal FI were normalized to respective background measurements. The right panel shows a histogram of cyst numbers at different ranges of FI, indicating changed distributions of FI in cysts due to the presence of the Mdr1 inhibitor verapamil. The gram was set using Microsoft Excel according to the data series of FI. Red lines show curves of the distribution changes.

**Supplemental Tables**

Table S1. TaqMan gene expression assay numbers for real-time PCR analysis

| Gene symbol | Gene name | TaqMan® ID |
| --- | --- | --- |
| *Gapdh* | glyceraldehyde-3-phosphate dehydrogenase | Rn99999916_s1 |
| *Slco1a2, (Oatp2)* | solute carrier organic anion transporter family, member 1A2 | Rn00756233_m1 |
| *Slc15a1, (Pept1)* | solute carrier family 15 (oligopeptide transporter), member 1 | Rn01466071_m1 |
| *Abcc2, (Mrp2)* | ATP-binding cassette, subfamily C (CFTR/MRP), member 2 | Rn00563231_m1 |
| *Abcb11, (Bsep)* | ATP-binding cassette, subfamily B (MDR/TAP), member 11 | Rn01515444_m1 |
| *Cftr* | cystic fibrosis transmembrane conductance regulator | Rn01455971_m1 |
| *Ggt1* | gamma-glutamyltransferase 1 | Rn00587709_m1 |
| *Aqp1* | aquaporin 1 | Rn00562834_m1 |
| *Gpbar, (Tgr5)* | G protein-coupled bile acid receptor 1 | Rn01400316_s1 |
| *Onecut1, (Hnf6)* | one cut homeobox 1 | Rn00575362_m1 |
| *Hnf4a* | hepatocyte nuclear factor 4, alpha | Rn04339144_m1 |

Table S2. List of antibodies used for immunocytochemistry analyses

| Antibodies | Catalog | Manufacturer | Dilution | Name | Species reactivity | Host species |
| --- | --- | --- | --- | --- | --- | --- |
| Albumin | MP55727 | [MP Biomedicals](https://www.google.com/url?sa=t&rct=j&q=&esrc=s&source=web&cd=1&cad=rja&uact=8&ved=0ahUKEwji6cuW7cPbAhUCCqYKHcvpCKwQFggpMAA&url=http%3A%2F%2Fwww.mpbio.com%2Fincludes%2Fmsds%2F0855727%2FMP_DS_0855727.pdf&usg=AOvVaw3rQZzATmEoG4yEd2fTIfyE) | 1:200 | Goat IgG Fraction to Rat Albumin | R | G |
| Mrp2 | [ab3373](http://www.abcam.com/mrp2-antibody-m2-iii-6-ab3373.html) | Abcam | 1:100 | Anti-MRP2 antibody [M2 III-6] | H, R | M |
| CK19 | [ab7755](https://www.abcam.com/cytokeratin-19-antibody-ba-17-ab7755.html) | Abcam | 1:500 | Anti-Cytokeratin 19 antibody [BA-17] | M, H, R | M |
| Krt19 | [NB100-687](https://www.novusbio.com/products/cytokeratin-19-antibody_nb100-687) | Nocusbio | 1:100 | Cytokeratin 19 Antibody | M, H, R | Rb |
| CK7 | [ab181598](https://www.abcam.com/cytokeratin-7-antibody-epr17078-ab181598.html) | Abcam | 1:500 | Anti-Cytokeratin 7 antibody [EPR17078] | M, R, H | Rb |
| Ae2 | [ab42687](https://www.abcam.com/ae2-antibody-ab42687.html) | Abcam | 1:200 | Anti-AE2 antibody | M, H, R | Rb |
| Aqp1 | [ab9566](https://www.abcam.com/aquaporin-1-antibody-122-ab9566.html) | Abcam | 1:200 | Anti-Aquaporin 1 antibody [1/22] | M, H, R | M |
| CFTR | ab2784 | Abcam | 1:400 | Anti-CFTR antibody [CF3] ab2784 | M, R, H | M |
| Anti-Rabbit IgG-TRITC | T6778 | Sigma-Aldrich | 1:200 | Anti-Rabbit IgG-TRITC | R | G |
| Anti-Rabbit IgG-FiTC | F9887 | Sigma-Aldrich | 1:200 | Anti-Rabbit IgG-FiTC | R | G |
| Anti-Nouse IgG-TRITC | T2402 | Sigma-Aldrich | 1:200 | Anti-Nouse IgG-TRITC | M | Rb |
| Anti-Mouse IgG-FITC | F9137 | Sigma-Aldrich | 1:200 | Anti-Mouse IgG-FITC | M | Rb |
| Anti-Goat IgG Alexa Fluor 488 | A21467 | Life technologies | 1:100 | Anti-Goat IgG Alexa Fluor 488 | G | C |

R: rat; M: mouse; Rb: rabbit; G: goat; C: chicken; H: human
